# Objective-lens-free Fiber-based Position Detection with Nanometer Resolution in a Fiber Optical Trapping System

**DOI:** 10.1038/s41598-017-13205-6

**Published:** 2017-10-13

**Authors:** Chaoyang Ti, Minh-Tri Ho-Thanh, Qi Wen, Yuxiang Liu

**Affiliations:** 1 Department of Mechanical Engineering, Worcester Polytechnic Institute, Worcester, MA 01609 USA; 2 Department of Physics, Worcester Polytechnic Institute, Worcester, MA 01609 USA

## Abstract

Position detection with high accuracy is crucial for force calibration of optical trapping systems. Most existing position detection methods require high-numerical-aperture objective lenses, which are bulky, expensive, and difficult to miniaturize. Here, we report an affordable objective-lens-free, fiber-based position detection scheme with 2 nm spatial resolution and 150 MHz bandwidth. This fiber based detection mechanism enables simultaneous trapping and force measurements in a compact fiber optical tweezers system. In addition, we achieved more reliable signal acquisition with less distortion compared with objective based position detection methods, thanks to the light guiding in optical fibers and small distance between the fiber tips and trapped particle. As a demonstration of the fiber based detection, we used the fiber optical tweezers to apply a force on a cell membrane and simultaneously measure the cellular response.

## Introduction

Optical tweezers have been widely used as a powerful and adaptive tool in biophysical studies^1–6^, biomechanical research^7^, tests of the fundamental natures of gravity^8^, and biochemistry^9^, thanks to their capability of manipulating micro/nanoscale particles and measuring nanometer scale displacements^10^. Traditionally, an optical trap is created by focusing a single laser beam with a high numerical aperture (NA) objective lens^11^. A particle with a refractive index higher than the medium can be trapped in the region with maximum light intensity.

Precise detection of the trapped particle position is crucial for trapping stiffness calibration. Video images acquired with a high NA objective lens and a camera have been used for position measurements with a resolution at the sub-pixel accuracy (typically 5 nm^10^ determined by centroid-finding algorithms^12–14^). However, in this camera based method, the detection bandwidth, which defines the temporal resolution of measurements, is limited by the image acquisition speed that is determined by the computer speed or memory capacity^15^, in addition to the camera frame rates. One can increase the frame rate of camera to improve temporal resolution, but at the expense of the signal to noise ratio, which decreases as the square root of the frame rate^10^. Currently the highest position tracking frequency with the camera based method is around 10 000 frame per second under a strong white illumination (a 100 W halogen lamp)^16^. Position sensitive detector (PSD) based method is an alternative position detection method with high spatial and high temporal resolution. Optical signal, which is either the scattering from the trapped particle or the interference between forward-scattered light from the particle and unscattered light^17–19^, is collected by an objective lens and detected by a PSD. The electrical outputs of the PSD allow one to measure the particle displacements. The PSD based method is more sensitive with sub-nanometer resolution^20,21^ and generally have higher bandwidth (more than 100 kHz)^10^ than the camera based method. However, both PSD and camera based measurements requires a high NA objective lens. Although used in most existing optical tweezers, objective lenses introduce intrinsic limitations in the optical tweezers system, such as bulkiness, short working distances, and difficulty to be integrated. These limitations may hinder their applications where the space is tightly confined or a trap is needed outside a lab environment, such as *in-vivo* measurements.

Here, we report an objective-lens-free, optical fiber based position detection mechanism with a displacement resolution of 2 nm on an inclined dual fiber optical tweezers platform. This allows one to deliver a miniaturized, self-sustaining fiber optical trapping system, in which a three-dimensional (3D) optical trap is created and calibrated *in situ* without the need of an objective lens. The fiber based position detection mechanism is based on differential optical signal that is scattered by the trapped particle, collected by the two fibers, and measured by a balanced photodiode. We achieved a high detection bandwidth (up to but not limited to 150 MHz, depending on the photodiode bandwidth) without the need of an objective lens, expensive high-speed camera, or external position measurement devices, such as a PSD. With the objective lens removed from the optical tweezers while the capability reserved for precise position detection, the fiber based detection mechanism combined with the inclined dual fiber optical tweezers is a unique and affordable tool that can be inserted in tightly confined space, trap single microscale particles, and apply or measure picoNewton-level forces both in laboratories and in the fields.

We experimentally verified the objective-lens-free, fiber based detection mechanism by comparing the trapping stiffness calibration results with those from camera based and PSD based detection methods. While the calibration results from all three methods are in good agreement, the fiber based detection mechanism demonstrated a much higher temporal resolution of 150MHz determined by the balanced photodiode. As a proof of concept of the uniqueness of the fiber based detection method, optical forces were applied to live cells, and real-time cellular responses were simultaneously measured without the need of objective lens. Therefore, the fiber based detection mechanism on the inclined fiber optical tweezers platform can be potentially used *in vivo* to characterize the mechanical properties of cells under the external stimuli. It is noted that there are some limitations of the fiber based detection. These limitations include the requirements of spherical particle shape, challenge to reach particles deeply embedded in a 3D solid matrix, position detection only in one dimension, and the fiber mechanical stability, all of which will be discussed further in the Discuss section. Even with these limitations, by removing the common need of a cumbersome high NA objective lens, the fiber based detection mechanism offers an insight into developing an integratable, portable, and miniature optical tweezers that can create and calibrate a 3D trap in the field.

## Results

### Principles of the fiber based detection mechanism

**Figure 1:**
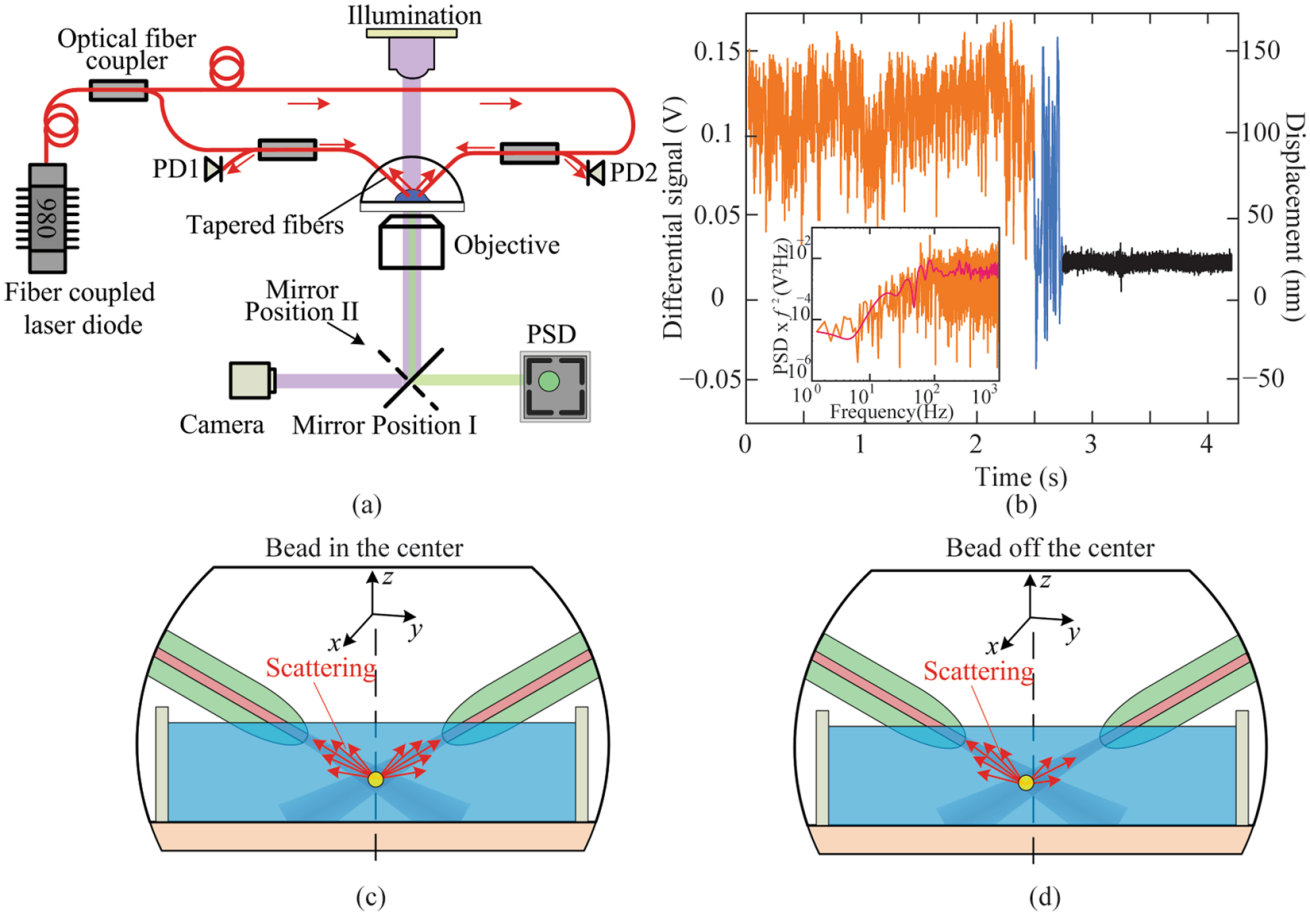
The setup, experimentally acquired optical signal, and working principle of the fiber based detection mechanism on the inclined dual fiber optical tweezers platform. (**a**) Schematic of the experimental setup of fiber optical tweezers with three detection methods (fiber, PSD, and camera based methods). PD1 and PD2 are the two optical inputs of the same differential photodiode. (**b**) Experimentally obtained data with the fiber based detection mechanism. The left vertical axis is the output of a differential photodiode, and the right is the corresponding displacement of a trapped 4.63-μm silica bead. The orange curve is the optical signal with a trapped bead, the black curve without a trapped bead, and the blue curve with the bead escaping from the trap when the DI water is moved fast. (Inset) The indirect method of measuring the displacement sensitivity (V/m) of the fiber based detection mechanism. The 2 nm resolution of the detection mechanism is determined by the root mean square of the noise signal (the black curve). (**c,d**) Schematics of the working principle of the fiber based detection mechanism. The scattered light collected by the two fibers are (**c**) the same when the bead is centered and (**d**) different when bead is off the center.

When the particle is located at the center of the trap (equilibrium position), there is no difference in the back-scattered optical power collected by the two fibers, as shown in Fig. 1c. When the particle moves to the left (along the *y* axis), the scattered optical power collected by the left fiber is higher than that by the right, and the difference in the collected optical power scales with the *y*-axis displacement, as shown in Fig. 1d, under the condition that the trapped particle lies in the linear range of differential signal versus particle displacement. Therefore, the *y*-axis particle position can be measured by the differential optical power collected by the two fibers. The differential optical signal is not influenced by the *x-* and *z*-axis positions, assuming small displacements. More details of the working principle can be found in the Supplementary information (Fig. S1a and S2).

To validate the fiber based detection mechanism, we incorporated traditional camera based and PSD based detection methods in the setup for comparison. The bead position is detected by a high-speed camera and a PSD through a high NA objective lens, in addition to by the two fibers. The light path of camera based detection and PSD based detection methods are shown in purple and green stripes in Fig. 1a, respectively.

### Results of the fiber based detection mechanism on the inclined dual fiber optical tweezers platform

The fiber based detection was carried out on the inclined dual fiber optical tweezers platform to demonstrate simultaneous particle trapping and calibration. Spherical silica beads with a diameter of 4.63 μm were used in the experiment. Once a silica bead was trapped by the inclined dual fiber optical tweezers, the back-scattered light from the trapped bead was collected by the same fibers and detected by a balanced photodiode. The experimentally measured differential optical signals with and without a silica bead in the trap are shown as blue and black curves in Fig. 1b, respectively. The short distance between the trap and each fiber tip (typically around 35 μm) allows the back scattered optical signal from the trapped bead to be efficiently collected by optical fibers, while removing the unwanted signal such as the ambient light and the laser scattered by particles not in the trap. As a result, a signal-to-noise ratio of 50:1 was achieved as shown in Fig. 1b. The position sensitivity of the objective-lens-free detection mechanism was obtained to be 0.64 V/μm with the indirect position calibration method^22^, as shown in the inset of Fig. 1b. The differential photodetector output with the unit of V can then be converted to the bead displacement with a unit of nm. The root mean square of the noise signal in the black curve is 2 nm, which is the resolution of the fiber based detection. The average signal of the black curve in Fig. 1b is not zero because the output optical power at the two fibers were not exactly equal and there might be minor fiber alignment errors.

The fiber based detection can be used to calibrate optical trap stiffness in an inclined dual fiber optical tweezers. The forces applied by the optical trap can be considered as restoring “spring” forces on the trapped particle. The particle thus undergoes a confined Brownian motion, monitoring which allows one to back out the trapping stiffness. In this work, we used three different position detection methods, namely fiber based, PSD based, and camera based methods, to obtain the position data of a trapped bead. These data were processed by the power spectrum analysis method^23–26^ (for the fiber based detection mechanism and PDS methods) and equipartition method^10,27–31^ (for camera based methods), in order to calibrate the trap stiffness. More details of three different position detection methods can be found in Methods section and Supplementary information. The comparison of the calibration results with the three position detection methods allows us to validate the fiber based detection mechanism.

**Figure 2:**
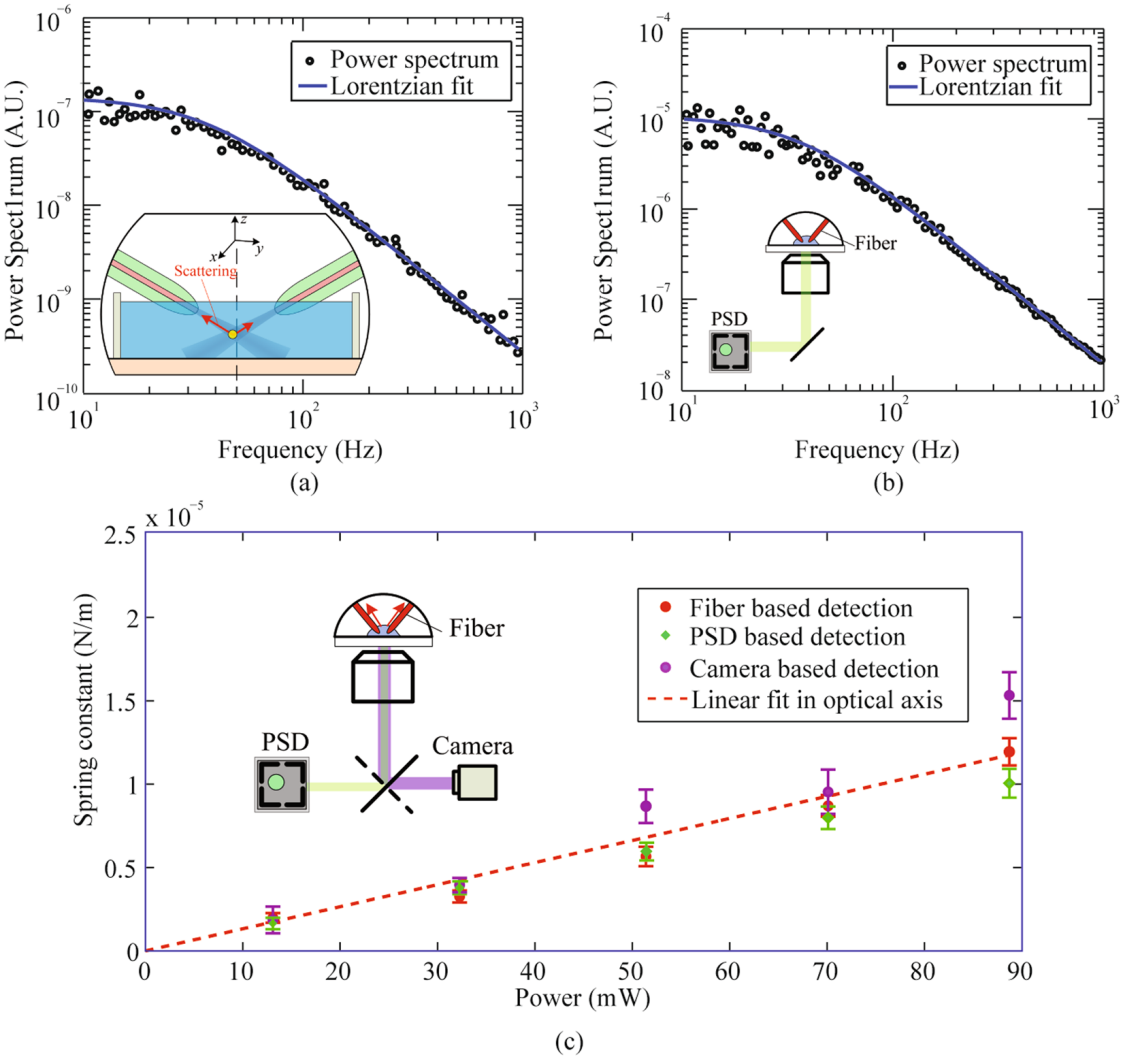
Verification of the fiber based detection mechanism by the PSD based detection method, via power spectrum analysis, and the camera based detection method, via equipartition theorem. Typical experimentally measured power spectrum data of a 4.63 μm bead trapped in water (black circles) and Lorentzian fitting (solid curves) from the fiber based detection (**a**) and PSD based detection method (**b**) at 69.6 mW in the *y* axis. (**c**) Optical trapping spring constant as a function of optical power, based on the fiber based detection (red dots), PSD (green squares), and camera based detection method (purple circles). The linear fitted curve (red dashed line) to a set of optical spring constants from the fiber based detection passes through point (0, 0). The optical power shown is the power emitted by each fiber.

In the experiment, the displacement of a trapped bead was detected simultaneously by the optical fibers and by a PSD (or camera) through the objective lens. In a PSD based detection, the obtained displacement power spectra were used to determine the corner frequency and, in turn, the optical trapping spring constants. Typical power spectrum data obtained with the fiber based detection and with the PSD method in the optical axis (*y* in Fig. 1c) are shown in Fig. 2a and b, respectively. The Lorentzian fitting of the power spectra provided corner frequencies ${f}_{0}$ of 35.9 ± 1.3 Hz with the fiber based detection (Fig. 2a) and 34.5 ± 1.4 Hz with the PSD detection (Fig. 2b). The corresponding optical trapping spring constants were 8.7 ± 0.3 pN/μm and 8.4 ± 0.3 pN/μm, respectively. The uncertainties were determined by the Lorentzian fit. It is noted that the bandwidth of fiber based detection is determined by that of the balanced photodetector, which in our setup is 150 MHz and can be readily improved by choosing different photodetectors.

We further investigated the dependence of optical trapping stiffness on optical power with the three methods. The experimental data are organized and shown in Fig. 2c. Each data point of PSD and fiber based detection in Fig. 2c was acquired from the Lorentzian fitting of a set of power spectrum data at a fixed power, while that of the camera based method was obtained from analyzing of ~25 000 subsequent microscopic images recorded by a high-speed camera. The measurements with all three methods agree well, and there is a linear relationship between the spring constant and the optical power. This result confirms the fiber based detection mechanism is a reliable method for optical trapping stiffness calibration.

The error bar of each data point in Fig. 2c is determined by the standard deviation of results from 5 independent measurements. The sources of the uncertainties arise from electronic noise, environmental noise, uncertainties in the bead diameter and temperature, uncertainties in the Lorentzian fitting, image quality, and the effects of finite sampling frequency during data acquisition process.

By comparing the results shown in Fig. 2c, we noticed that the camera based measurements are more scattered around the fitted linear progression and that the fiber based detection results are least scattered. One possible reason is that the *z*-axis equilibrium position (see Fig. 1a) of the trapped bead depends on optical powers. This *z*-axis position change could vary the particle position detected by the objective lens based methods, i.e., PSD and camera based methods, which could introduce nonlinearity between the spring constant and optical power. By comparison, differential signals collected by optical fibers are not affected by the *z*-axis position change, resulting in a more linear stiffness-power curve. In addition, the trapped particle is farther away from the objective lens compared with the fiber tip, so the PSD detection and camera based detection methods are more susceptible to optical scattering in the medium. Besides, the required optical filters for camera based detection as well as the fine adjustment of objective lens make the system of PSD and camera based detection much more complex, which may hinder the applications in a standard biological laboratory. By comparison, the fiber based detection mechanism provides more reliable measurements with a much more compact and straightforward experimental setup for optical trapping stiffness calibration.

### Combined fiber based trapping and position detection on the inclined dual fiber optical tweezers platform for cell mechanics study

The fiber based detection allows us to deliver a compact, easy-to-use, *self-sustaining* fiber optical trapping system, in which a three-dimensional (3D) optical trap is created and calibrated *in situ* without the need of objective lens. The unique capabilities of this system makes it a potential tool for cell mechanics study without occupying the objective lens.

As a demonstration, we used the fiber based detection combined with the inclined dual fiber optical tweezers for controlled force application to a cell and simultaneous cell response measurements. Specifically, an optical force was applied on a bead attached to a fibroblast membrane by the inclined dual fiber optical tweezers. The cell developed an internal force in response. Such response was manifested as the local stiffness change of the cell membrane^32,33^. The fiber based detection allows the real-time monitoring of the bead motion and hence the measurement of the fibroblast stiffness.

**Figure 3:**
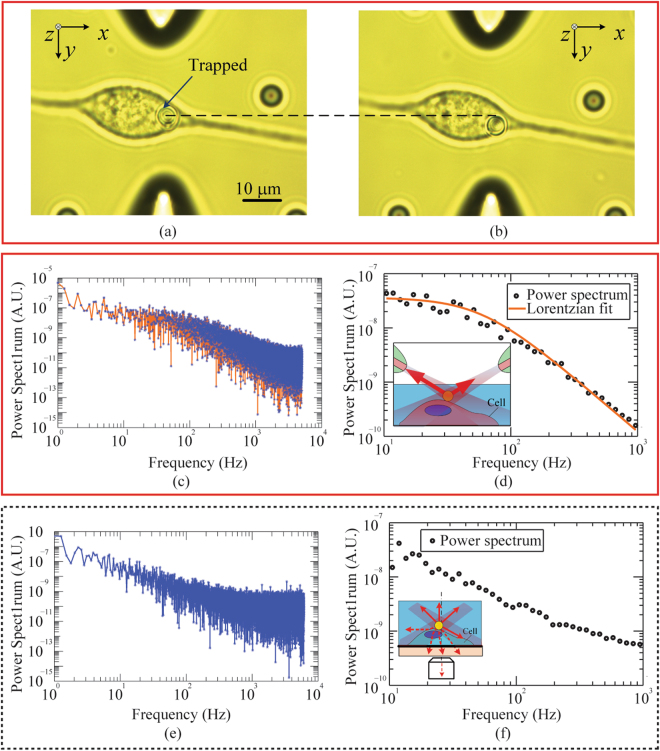
The fiber based detection combined with the inclined dual fiber optical tweezers for cell mechanics study. (**a**) A trapped bead was moved to a selected location on the cell surface with no external force applied. The bead was held in place to be spontaneously bonded to the cell surface. (**b**) The bead was moved in the *y* direction by displacing the inclined dual fiber optical tweezers to apply an external force to the cell membrane. (**c–f**) Power spectrum data of the bead. Raw data (**c**) obtained with the fiber based detection in the *y* axis were blocked and fitted (**d**) by a Lorentzian. Raw data (**e**) obtained with a PSD though an objective lens were blocked (**f**) but cannot be fitted by a Lorentzian. Inset in (**d**): schematics showing back-scattered signal collected by the fibers without optical scattering and distortion by cells; Inset in (**f**): schematics showing the optical distortion and scattering by cells in the transmission light detected by the objective and PSD.

In the experiment, a drop of diluted bead solution was added into the cell culture medium containing 3T3 fibroblast cells. An uncoated 4.6-μm silica bead was trapped by the inclined dual fiber optical tweezers and moved to a selected location on a cell surface, shown in Fig. 3a. The bead, serving as a force handle, was spontaneously bound onto the cell surface after being held in place for 10 seconds. An optical force was applied on the cell by displacing both fibers relative to their initial positions, as shown in Fig. 3b. The direction and magnitude of the force was controlled by the position of the bead relative to the trap center. The thermal motion of the bead was simultaneously monitored by the fiber based and PSD based detection methods.

The experimentally obtained power spectrum data of the bead are shown in Fig. 3c–f. The raw data obtained by the fiber based and PSD based detection are shown in Fig. 3c and e, while the blocked data are shown in Fig. 3d and f, respectively. The power spectrum from the fiber based detection was fitted by a Lorentzian. The corner frequency obtained from the bead on the cell surface is 62.9 ± 3.9 Hz (Fig. 3d), while that in water is 35.9 ± 1.3 Hz (Fig. 2a). This increase in corner frequency rises from the mechanical stiffness of the cell membrane.

It is noted that Lorentzian fitting fails for the power spectrum obtained by the PSD, as shown in Fig. 3f. This might be caused by the optical scattering and distortion due to the subcellular structures, since the signal detected by the PSD is the light transmitted through the cell, as shown in the inset of Fig. 3f. By comparison, the light collected by the optical fibers is back-scattered from the bead and hence less influenced by the subcellular structures, as shown in the inset of Fig. 3d. Besides, the back-scattered light from subcellular structures (noise) are blocked by the bead before received by both fibers, thanks to the low NA of tapered optical fibers (≈0.4), which helps to enhance the signal to noise ratio (SNR) for the fiber based detection mechanism. The fitting failure of PSD based method proves that fiber based detection is more robust and reliable for studies of biological cells, thanks to its robustness to the optical noise induced by the cell scattering.

Real-time monitoring cellular response to external stimuli, with a high temporal resolution, is important to study and measure the evolution of cell mechanical properties over time. Biological processes of cells, such as cell division and differentiation^34^, have been shown to be associated with the cell response to external cues in their environment, which in turn change cell mechanical properties^35^. In this work, the fiber based detection on the inclined dual fiber optical tweezers platform was used to measure real-time cell stiffness changes in response to external mechanical stimuli. First, a silica bead without coating was trapped and moved onto cell surface to allow spontaneous bonding. We moved the inclined dual fiber optical tweezers in the *y* direction with a controlled distance (~ 1.6 μm), in order to apply a force to the cell membrane. During this process, the optical power was kept to be constant. The corner frequency of the bead was determined by both the optical trap and cell stiffness. Since the optical trap stiffness is constant with a fixed optical power, the corner frequency was monitored as a measure of cell response.

**Figure 4:**
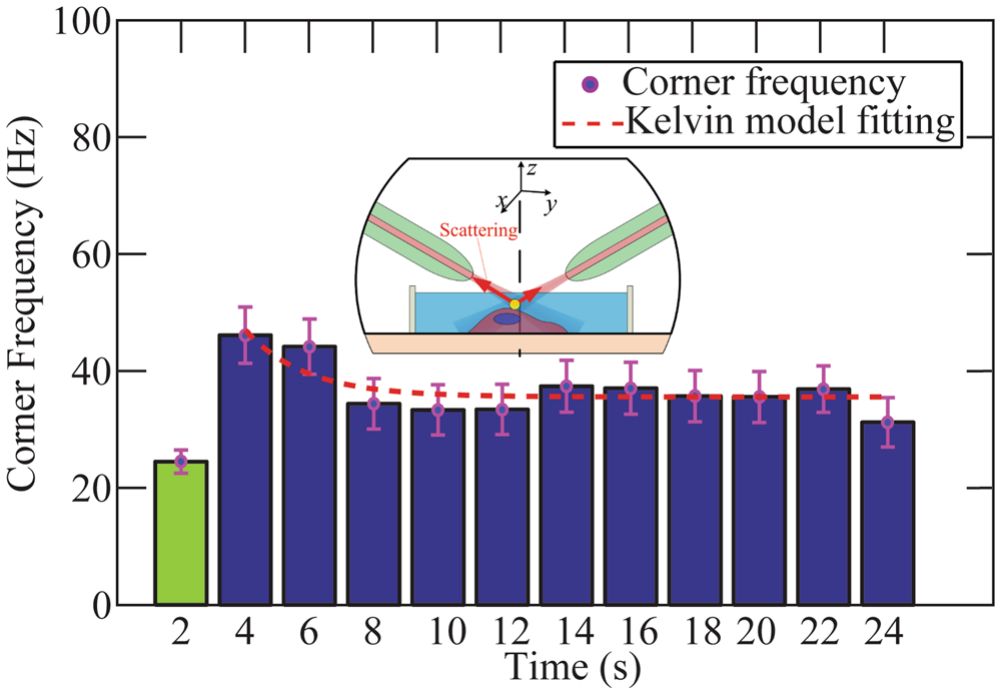
Cell response in the corner frequency monitored in real time. The green bar represents the measured corner frequency when there was no force applied to the cell, serving as a reference. A constant force was applied to cell membrane at 2 seconds, and the blue bars are the measured corner frequencies after the force application. The change of the blue bars indicates the effective stiffness change of the cell membrane, which is the cell response to the external mechanical stimulus. The red dashed curve is the fitting based on the Kelvin model, which describes the creep behavior of viscoelastic materials. Inset: schematic of fiber based trapping and detection mechanism that was used for external force application and cellular response monitoring.

The corner frequency before (green) and after (blue) a force was applied are shown in Fig. 4. Each bar represents the corner frequency obtained from raw data recorded within 2 seconds, the time coordinate of each bar is the end of the recording period. The uncertainty in corner frequency is determined by the 95% confidence interval range of the Lorentzian curve fitting. We observed that the measured corner frequency increased immediately after the external force was applied at 2 seconds, followed by a graduate decrease, and was stabilized at around 14 seconds.

The observed data in Fig. 4 can be explained in the following. When the cell is deformed by the external force applied through the bead, the cell responded immediately by increasing its membrane stiffness. This may be because the bead-induced cell deformation stiffens the internal cellular structures^32,33^. A relaxation response of the cell membrane was followed by a slowly decreasing of the stiffness. The Kelvin model of viscoelastic materials (red dashed curve in Fig. 4) was used to describe this creep behavior of cell^36,37^.

## Discussion

We developed an objective-lens-free, fiber based position detection mechanism that can be used to measure the trapped particle displacement with nanometer spatial and a hundred MHz temporal resolution. It allows us to deliver an inclined dual fiber optical tweezers platform that can realize 3D optical trap and optical stiffness calibration *in situ* without the requirement of objective lens. Combined with the fiber based position detection mechanism, the inclined dual fiber optical tweezers are a compact, easy-to-use, self-sustaining fiber optical trapping system that can be used in applications in tightly confined space as well as cell mechanics study. In addition to the advantages mentioned above, the fiber based position detection mechanism has the benefit of less influence from the optical scattering and distortion by subcellular structures, which is particular useful in cell mechanics study. As a proof of concept, we used the fiber based position detection on the inclined dual fiber optical tweezers platform to measure the real-time fibroblast stiffness changes in response to a static mechanical stimulus.

Although in this work, the experiments of the combined fiber based detection with the inclined dual fiber optical tweezers were still carried out on a microscope platform, the objective lens was not used to create or measure the optical trap. The inclined dual fiber optical tweezers can serve as a building block that can be mounted on regular microscope for cell mechanics study, while the objective lens is freed up for other applications such as traction force microscopy.

In the experiment, the inclined angle θ and the distance between two fiber tips are important for both optical trap and position detection. θ is defined as the angle between a fiber and the vertical direction, and two fibers share the same θ value. θ can influence the trapping efficiency, trap stability, and minimum optical power needed to realize the 3D trapping. According to our previous work^38^, the minimum θ value for 3D trapping is around 50°. It is noted that the difficulty of alignment for realizing a 3D trap increases when the inclined angle is larger than 70°, with the most difficult alignment at an inclination angle of 90°, i.e., with the two fibers along a straight line. The inclination angle is less critical for the fiber based detection mechanism. In general, a larger inclination angle and smaller fiber separation can enhance the position sensitivity. However, when θ is close to 90°, each fiber collects forward-scattered light from the other fiber, in addition to the back-scattered light from itself. Due to this coupling effect, the linear relationship between collected optical power and displacement might not be valid, resulting in failure of the fiber detection when θ is close to 90°. Considering the trapping efficiency, trapping stability, and alignment difficulty as well as the detection sensitivity, we set the inclined angle and the distance between two fibers to be 55° and 80 μm, respectively.

Since single fiber detection has been used by previously published work^39,40^, we would like to emphasize that the dual fiber detection mechanism can measure the mechanical motion of the trapped particle along one direction, not coupled with the other two. This one-dimensional measurement is particularly useful for anisotropic media or particle motion. By rotating the orientation of the fiber based detection setup, motion along other directions can be measured separately. By comparison, the 3D motion is coupled in the measurements of single fiber detection, and it is challenging to decouple one from the other two.

It is worth noting that the fiber based detection mechanism might be influenced by particle shapes, which might affect the accuracy of the position detection. If the particle shape is not spherical, the scattered light is not only dependent on the particle displacement but also on the orientation. It is also challenging for particles that are deeply embedded in a 3D solid matrix, since the two fiber tips are relatively close (tens of micrometers) to the trapped particle in the experiments. It is preferred if a stiffer fiber holder is used, which can improve the fiber mechanical stability and reduce the influence of fiber motion on the position detection. The current setup can be used to detect displacement along one dimension, which is beneficial for anisotropic media or motion, and it is challenging to decouple the other two-dimensional motion from fiber based detection signals. Position detection along more than one direction can be realized by rotating the detection system, which is based on fiber and can be readily rotated.

To make the fiber based detection more reliable and easier to use, a modular system development is underway. Currently, the two fibers were separately held by two sets of mechanical stages, which are in turn held by a common board in the current experimental setup. This setup allows easy change of the system parameters, such as the fiber inclination angle and fiber separation. However, these multiple movable parts are subject to mechanical drift. We are currently working on a modular system, where both fibers are aligned and fixed on a common board with no movable parts. The purpose of developing the modular trapping system is to make the fiber based detection mechanism accessible to anybody, who might not know how to set up and align the fibers.

## Methods

### Prototype design and experimental setup

The fiber based position detection mechanism on the inclined fiber optical tweezers platform is designed based on the dual fiber optical tweezers, which can be found in our previous work^38,41,42^. Briefly, the fiber based position detection mechanism, as well as the inclined fiber optical tweezers, was set up on a microscope platform. Light from a fiber-coupled 974 nm laser diode (AC 1405-0400-0974-SM-500, Eques) was split into two lensed fibers (TLF SM1060, Nanonics Imaging) through a 3dB coupler (22-12798-50-23162, GouldFiber Optics), as shown in Fig. 1a. All the fibers in the system were single-mode at 974 nm. The two light beams emitted from the lensed fiber tips can three-dimensionally trap microscale particles in water close to the beam intersection. The back-scattered light by the bead was collected by the two lensed optical fibers and measured by two inputs (PD1 and PD2) of a balanced photodiode (PDB450C, THORLABS). The differential output from the photodiode enabled the measurements of bead positions with nanometer resolution. The forward-scattered light collected by an objective lens was recorded by either a PSD (DL100-7-PCBA3, First Sensor) or a high-speed camera (iXon 3 EMCCD, Andor).

### Cell Culture

NIH-3T3 fibroblasts were cultured in Dulbecco᾿s modified Eagle᾿s medium (DMEM) supplemented with 10% bovine calf serum, 100 Units/ml potassium penicillin, 0.1 mg/ml streptomycin sulfate, and 1mM L-glutamine.

### Preparation of particle solution

Silica beads (Bangs Laboratories, Inc.) with a diameter of 4.63 μm and density of 2.0 g/cm^3^ were used for the experiments. A diluted bead solution, with a ratio of deionized (DI) water to original bead solution (10.2%, 0.5 g in weight) of 6000:1, was ultrasonicated in order to reverse bead aggregation. The prepared bead solution (~2 ml) was then added on a coverslip, where the trapping experiment was carried out. To prevent the water on the coverslip from drying up under the illumination light and air flow, DI water was added to the coverslip frequently^38^.

### Calibration methods

All three detection methods used in the position calibration are detailed below.

### Power spectrum analysis method

In order to calibrate the trapping stiffness, experimental data was processed by the power spectrum analysis method for the fiber based detection and the PSD based detection. In the power spectrum analysis, the motion of a particle in a harmonic potential can be described by^23^:

1 $$m\ddot{x}(t)+\gamma \dot{x}(t)+{k}_{x}x(t)={(2{k}_{B}T{\gamma }_{0})}^{1/2}\eta (t),$$

and the one-sided power spectrum of the particle displacement can be expressed as^2,41^

2 $${S}_{xx}(f)=\frac{{k}_{B}T}{{\pi }^{2}\gamma ({{f}_{0}}^{2}+{f}^{2})},$$

where *k*_*B*_ is Boltzmann’s constant, *T* is the absolute temperature, and *γ*is the hydrodynamic drag coefficient of the object ($\gamma =6\pi \eta a$ for Stokes drag on a sphere with a radius *a* in a medium with a viscosity$\eta$). ${f}_{0}$ is the corner frequency or roll-off frequency, which is the characteristic of the fitted experimental curve, and provides the trapping spring constant *k* by

3 $$k=2\pi \gamma \,{f}_{0}.$$

The power spectrum method does not require the detector displacement sensitivity for calibration.

### Equipartition theorem

Equipartition theorem was used for the camera based detection. Equipartition theorem assumes $\frac{1}{2}{k}_{B}T$ as the thermal energy for each degree of freedom of the particle motion, where ${k}_{B}$ is the Boltzmann constant (≈1.38×10^−23^ JK^−1^) and $T$ is the absolute temperature. On the other hand, this thermal energy is associated with thermal fluctuations of the particle in an optical trap with stiffness ${k}_{y}$ in *y* direction can be described by:

4 $$\frac{1}{2}{k}_{y}\langle {y}^{2}\rangle =\frac{1}{2}{k}_{B}T$$

The equipartition method requires a calibrated position sensing device, but does not require pre-known viscosity of the trapping medium. The particle position used in the equipartition was measured by a high-speed camera, specified below.

### Image correlation algorithm used in camera based detection

**Figure 5:**
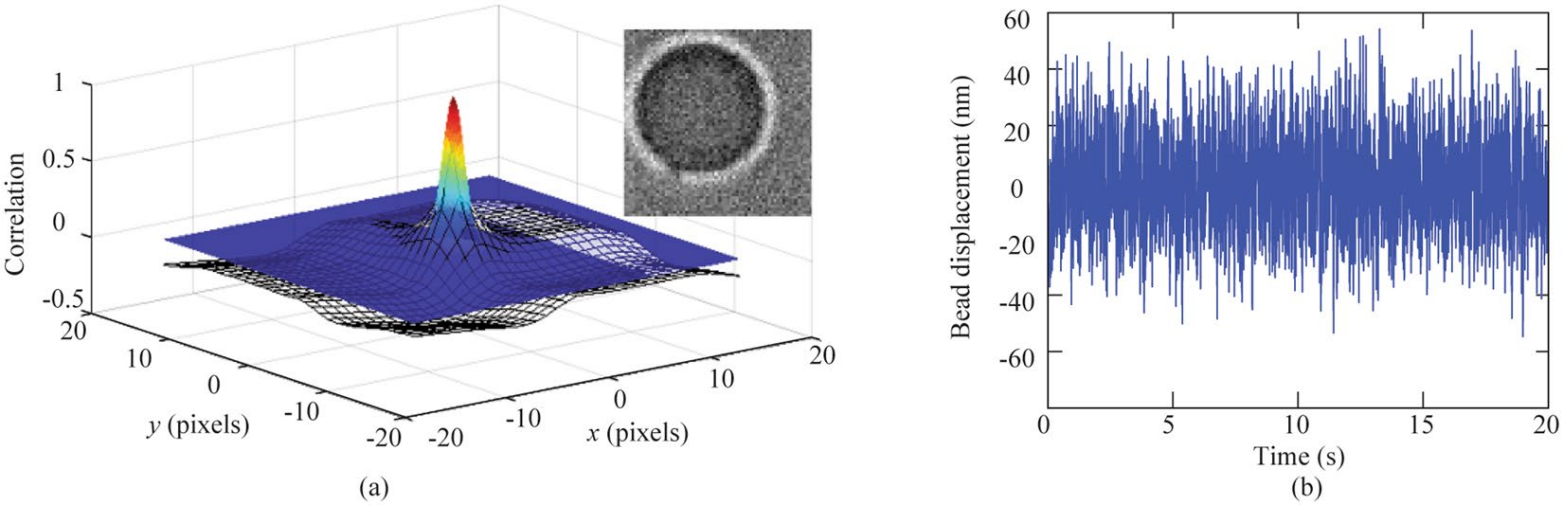
(**a**) 3D plot obtained with image processing showing the cross-correlation matrix value (mesh grid) overlaid with the fitted Gaussian function (color). The peak position of the Gaussian function is used to determine the bead displacement. Inset: A typical bright field image of the bead. (**b**) Time-domain position of the trapped bead obtained with the image processing. The trapping laser power was 51 mW.

In the camera based detection, bright field images (Fig. 5 inset) of the bead were obtained by an Andor iXon 3 EMCCD camera. Working at the “cropped sensor mode”, the camera took  64x64 pixel images at a frame rate of 248 frames per second. For each trapping power, five series of 5000 images were collected.

The time-lapse images were analyzed using a custom-built script in Matlab (Mathworks, Natick MA) to extract the centroid of the bead as a function of time. Each image was cross-correlated with the first image using a built-in Matlab function *normxcorr2*, which computes the normalized cross-correlation of two matrices. The resulting correlation matrix was fitted to a 2D Gaussian function, allowing the peak to be determined with subpixel accuracy. The peak position of the Gaussian function was then used to denote the position of the bead relative to its position in the first image. Figure 5a shows the cropped correlation matrix overlaid with the 2D Gaussian function. Finally, the low-frequency drift of the bead position was removed using a 6^th^-order high-pass Butterworth filter. Figure 5b shows the relative position of the bead after filtering.

## Electronic supplementary material

Objective-lens-free Fiber-based Position Detection with Nanometer Resolution in a Fiber Optical Trapping System

Brightfield image of the bead with size of 4.64 μm overlaid with the detected bead boundary
