# Supplementary material for: Objective-lens-free Fiber-based Position Detection with Nanometer Resolution in a Fiber Optical Trapping System
Source: Sci Rep. 2017 Oct 13;7:13168. doi: 10.1038/s41598-017-13205-6 (PMC5640681; doi:10.1038/s41598-017-13205-6)
Supplement: Supplementary file 1 — Objective-lens-free Fiber-based Position Detection with Nanometer Resolution in a Fiber Optical Trapping System [file 41598_2017_13205_MOESM1_ESM.pdf]

Supplementary information

Objective-lens-free Fiber-based Position Detection with Nanometer Resolution in a Fiber Optical Trapping System

Chaoyang Ti[1], Minh-Tri Ho Thanh[2], Qi Wen[2], and Yuxiang Liu*[1]

[1]Department of Mechanical Engineering, Worcester Polytechnic Institute, Worcester, MA, 01609, USA

[2]Department of Physics, Worcester Polytechnic Institute, Worcester, MA, 01609, USA

*Corresponding: yliu11@wpi.edu

Mathematical proof of the fiber based position detection mechanism. Objective-lens-free, fiber based position detection mechanism depends on the difference in the scattered optical power collected by the two fibers. Here we prove that the differential signal from those two fibers is linearly proportional to the trapped bead displacement, assuming small displacements. Optical beams emitted from both fibers are scattered by a trapped bead and gathered by both fibers. We will use P_{ij} (i, j = 1, 2) to represent the light emitted from Fiber i and collected by Fiber j. The scattered signal gathered by each optical fiber can be separated into two parts, one being light emitted from itself, P_{11} or P_{22}, and another being light emitted from the other fiber, P_{21} or P_{12}, as shown in Fig. S 1a. As a result, the total scattered optical power received by Fiber 1 (P_1) and Fiber 2 (P_2) can be described as:

$$P_1 = P_{11} + P_{21}, P_2 = P_{12} + P_{22}. \tag{1}$$

The influence of the bead displacement on the collected power is dependent on the moving direction. That is, the influence of the bead movement along the fiber axis on the collected power is different from that of the bead movement perpendicular to fiber axis. Therefore, we define the ratio of collected optical power to displacement as a displacement sensitivity. There exist 6 displacement sensitivities with a unit of W/μm: $D_\perp$, $D_\perp'$, $D_\perp''$, D_{II}, D_{II}', and D_{II}'', where the subscripts specify whether the direction of the bead displacement is parallel or perpendicular to the collection fiber axis. $D_\perp$ and D_{II} are the sensitivities of light emitted and collected by the same fiber, $D_\perp'$ and D_{II}' are those of light emitted by Fiber 1 and collected by Fiber 2, and $D_\perp''$ and D_{II}'' are those of light emitted by Fiber 2 and collected by Fiber 1. The detailed geometry diagram is shown in Fig. S 2. The dependence of P_{11} and P_{12} on the x-, y-, z-axis bead displacement can then be expressed as:

$$\begin{aligned} P_{11\_y} &= -D_\perp |y| \cos\theta - D_{II}\, y \sin\theta \\ P_{11\_x} &= -D_\perp |x| \\ P_{11\_z} &= -D_\perp |z| \sin\theta + D_{II}\, z \cos\theta \end{aligned}, \tag{2}$$

$$P_{12\_y} = -D_{II}' y \sin\theta \cos(2\theta - 90°) + D_{II}' \sin\theta \cos(180° - 2\theta) y$$
$$+ D_{\perp}' y \cos\theta \cos(2\theta - 90°) - D_{\perp}' y \cos\theta \cos(180° - 2\theta)$$

and

$$P_{12\_x} = -D_{\perp}'|x| \qquad (3)$$
$$P_{12\_z} = D_{II}' z \cos\theta - D_{\perp}|z|\sin\theta$$

P_{21} and P_{22} are:

$$P_{21\_y} = -D_{II}'' y \sin\theta \cos(180° - 2\theta) - D_{II}'' \sin\theta \cos(2\theta - 90°) y$$
$$- D_{\perp}'' y \cos\theta \cos(2\theta - 90°) - D_{\perp}'' y \cos\theta \cos(180° - 2\theta)$$
$$P_{21\_x} = -D_{\perp}''|x| \quad ; \qquad (4)$$
$$P_{21\_z} = D_{II}'' z \cos\theta - D_{\perp}|z|\sin\theta$$

and

$$P_{22\_y} = -D_{\perp}|y|\cos\theta + D_{II} y \sin\theta$$
$$P_{22\_x} = -D_{\perp}|x| \quad . \qquad (5)$$
$$P_{22\_z} = D_{II} z \cos\theta - D_{\perp}|z|\sin\theta$$

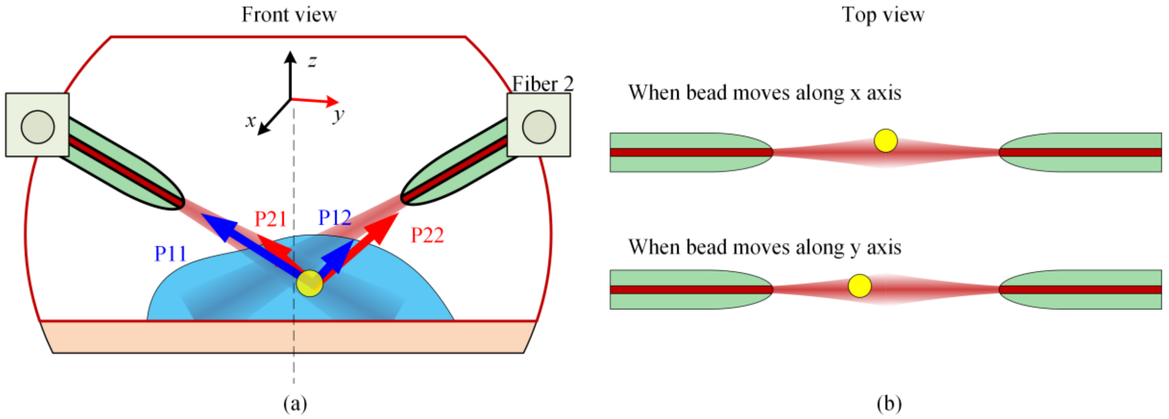

S 1. schematics of principle of fiber based detection mechanism (a) and top view (b). P_{11} and P_{12} are the light emitted from Fiber 1 and collected by Fibers 1 and 2, respectively. P_{21} and P_{22} are the light emitted from Fiber 2 and collected by Fibers 1 and 2, respectively.

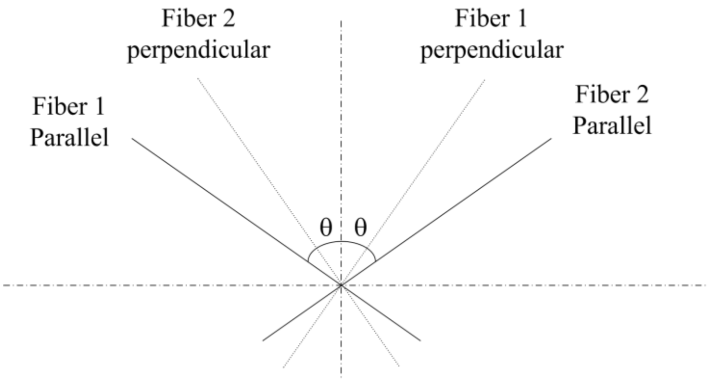

S 2. The detailed geometry diagrams.

Based on the above deductions, we can obtain the resultant differential power signal between two fibers as:

$$P_1 - P_2 = Cy \qquad (6)$$

Where *C* in Equation (6) stands for a constant and can be described as:

$$
\begin{aligned}
C = & -2D_{II}\sin\theta \\
& + \begin{Bmatrix}
(-D_{II}'-D_{II}'')\sin\theta\cos(180° - 2\theta) \\
+ (D_{II}'-D_{II}'')\sin\theta\cos(2\theta - 90°) \\
+ (-D_{\perp}'-D_{\perp}'')\cos\theta\cos(2\theta - 90°) \\
+ (D_{\perp}'-D_{\perp}'')\cos\theta\cos(180° - 2\theta)
\end{Bmatrix}
\end{aligned}
\tag{7}
$$

Equation (6) indicates that the differential power collected from two fibers is linearly proportional to the *y*-axis bead displacement, but not dependent to the *x*- and *z*-axis displacement. Therefore, we conclude that the differential power signal can be used to measure the *y*-axis bead displacement when the bead displacement is small.

Video Brightfield image of the bead with size of 4.64 μm overlaid with the detected bead boundary.
